# Dental and Facial Evidences of Constitutional Defect

**Published:** 1896-05

**Authors:** Eugene S. Talbot

**Affiliations:** Chicago, Ill.


### International Dental Journal.

Vol. XVII. May, 1896. No. 5.

### Original Communications.[1]

### DENTAL AND FACIAL EVIDENCES OF CONSTITUTIONAL DEFECT.[2]

BY EUGENE S. TALBOT,[3] M.D., D.D.S., CHICAGO, ILL.

Every observant practitioner has noted the contrasted temperaments of his patients, their varying mental and physical characteristics, their differing resistance to pain and disease, and their response to treatment. Certain cases occur to every dentist in which local states do not explain the facts. In the same family it has been noticed that while with one child eruption of the teeth is marked by no extraneous morbid phenomena, in another convulsions and, perchance, death result. In adults tooth-extraction has been noted to cause fatal hemorrhage, and in children gum-lancing has had the same effect. Enamel and dentine discolorations, varying from whitish brown to black, occur without local cause. Teeth normally developed display erosions and abrasions in places where friction from occlusion is impossible. This is especially noted in ataxics, paretic dements, and victims of constitutional dyscrasia.

---

[1] The editor and publishers are not responsible for the views of authors of papers published in this department, nor for any claim to novelty, or otherwise, that may be made by them. No papers will be received for this department that have appeared in any other journal published in the country.

[2] Read before the American Academy of Dental Science, Boston, February, 1896.

[3] Fellow of the Chicago Academy of Medicine.

Lipomatosis, or morbid corpulency, an expression of the gouty diathesis in children, for example, is often attended by jaw and tooth deformities, irritability, suspiciousness, capriciousness, and lack of will-power. The degenerate classes (congenital blind, congenital deaf-mute, epileptics, neuropaths, one-sided geniuses, hysterics, lunatics, paupers, prostitutes, criminals, etc.) are more susceptible to disease and have greater mortality than normal persons. The principle underlying this fact was recognized by Hippocrates twenty centuries ago, who also pointed out that even normal persons react differently to disease. In one the disease expends itself in pyorrhœa, in another gout of the great toe, in a third it appears as catarrh, in a fourth as biliousness, and in a fifth as mental depression. These constitutional variations may reveal themselves in local expression, as in supernumerary and deficient tubercles and teeth, variations in jaw size, in septum deflection, in turbinated bone, and in mucous membrane hypertrophy, with resultant adenoid vegetation.

Miller, of Berlin, deserves all possible honor for his clear demonstration of tooth decay in its primary etiology. It is a trite but somewhat defective truism that to know the cause is to know the remedy. The truism is defective in not recognizing any but an immediate cause. Behind this last, operate the more important remote or predisposing factors. The germ must have its culture medium. The immediate cause alone does not explain tooth-decay in constitutional disease in pregnant women, in school overstrain, and in the climacteric period between forty and fifty years of age in both sexes, etc. In those states oral antisepsis, hygiene, and prosthesis alone fails. Recent researches anent acromegaly certainly demonstrate that constitutional factors underlie tooth and bone-growth, and give a logical developmental explanation for what many dentists have clinically found to be a fact. While constitutional physical expressions of defect are often recognized at birth, the mental are not observed until later in life. Various tests are laid down for determination of deficiencies. Lombroso and Dana lay down the very broad rule that children who differ from their parents are of necessity degenerate. This claim is entirely too sweeping. The parents are the expression of many generations. A child may be perfectly healthy, yet present all the characteristics of grandparents; in other words, be an atavism, or "throw back." The mental state of the mother during pregnancy must be accepted as a factor, despite its denial by a few belated followers of Weissman, who himself has lately admitted maternal nutrition as an influence in variation. This factor explains the birth of several healthy children followed by the birth of a degenerate with a club-foot, cleft spine, etc. Again, children who resemble either one or the other parent are not infrequently degenerates. A dietetic fad for pregnant women propagated by a Hahnemanniac, now a faith-healer, produced what some mothers called "fruit diet" babies,—irritable, feeble defectives, in marked contrast with previous children. The women pregnant during the siege of Paris found the child born thereafter so uniformly defective that, among the middle and working classes, "child of the siege" was a synonyme for "doomed child." These children had enlarged thyroids, cleft palates, cleft spines, irregularities of the jaws, teeth, ears, and eyes, as well as decided susceptibility to nervous and other constitutional diseases.

The farmer who desires certain results not only looks after his seed, but the soil in which it is placed. There has long been a dental bias to regard the seed (the local manifestation) as the only thing of importance, while the soil (the constitution) can be safely disregarded. There is, however, a growing tendency among all specialists to examine into the state of the body as a whole. The eye surgeon takes into account gouty and rheumatic states, not to speak of born defects. The gynæcologist does the same. The surgeon looks cautiously into the general health before performing grave operations. The dentist, warned by his encounters with the bleeder diathesis, is beginning to do the same. Underlying many of these constitutional states which are the sources of such dangers, noticeably the "bleeders," is the condition known as degeneracy. The term of late has become popularized, and therefore needs definition. A degenerate, scientifically, is a person whose brain and nervous system is unstable from inherited or acquired taint in the parents, who has in consequence undergone imperfectly the embryologic changes to a higher type in tissues or organs, and therefore exhibits tendencies liable to extinguish the race, as a type, under the usual conditions of the struggle for existence. While the majority of alienists, embryologists, and teratologists have recognized this state for decades, the science of the subject was first formulated half a century ago by Morel, of France, and has since been carried to its greatest and, it must be admitted, rather unjustifiable extreme by Lombroso of Turin and his followers. To Lombroso's industry in collection of facts the world owes a debt which outweighs any evil resultant on his rather rash generalizations. The degenerate, according to Morel, exhibits various physical abnormalities which were supposed to be marks of heredity, and to which he gave the name "stigmata," or signs of degeneracy. The bones are, as to growth and development, most completely under the control of the nervous system. An osseous system normally developed usually denotes (other things being equal) a good physique. When the parts are not in harmony, "stigmata" of degeneracy appear. The stigmata of other systems are not so easily detected.

Lombroso claims that these stigmata so mark the degenerates, as a class, that, while they depart widely from the parental race type, they so approximate each other as to constitute a type, "the degenerate," which is a constant variety. While there is a certain truth in this, still the position of Lombroso is too absolute, since stigmata occur in normal persons as expressions of factors outside the field of degeneracy.

Stigmata of degeneracy may be confined to either the nervous or general system, while the other may not be materially affected. A congenital deaf-mute may be of fairly developed physique, while badly deformed individuals may have normally developed brains. In more marked degenerates (idiots, epileptics, prostitutes, and periodical drunkards) every part of the body may be more or less affected. These data are of special importance as clearing the ground for proper discussion of facts.

The higher and older the civilization the greater the evident degeneracy. In European countries more stigmata are observed than in this country; in New England more than in the West. More stigmata occur among the nobility of Europe than among the general population; the same is true of the American "society" class.

More investigations have been made and more has been written upon this subject of degeneracy by the French, Italians, Scandinavians, Germans, and Americans than by the English. Nothing appears in dental literature on this subject except so far as the dental arches are concerned. With this relationship dentists are chiefly concerned, and, the ground having been cleared, it is here fitting to discuss from a dental stand-point the various lesions of the oral cavity in their relation to systemic conditions. The following table by Moreau,[1] the alienist, outlines the classification of conditions more clearly than anything that has come under observation.

---

[1] Morbid Psychology.

### TABLE A.

NERVOUS AND HEREDITARY CONDITIONS OF IDIOSYNCRASIES.

| | | |
|---|---|---|
| Exceptional intelligence | Science. | |
| | Arts. | Music. |
| | | Painting. |
| | Letters. | |
| Lesions of the nerve-centres. | Brain. | Paretic dementia. |
| | | Paralyses. |
| | | Tumors. |
| | | Brain softening. |
| | | Congestion. |
| | Spinal cord. | Softening of spinal cord. |
| | | Myelitis. |
| Neurosis | Epilepsy. | |
| | Chorea. | |
| | Stammering. | |
| | Convulsive fits. | |
| | Hysteria. | |
| Intellectual | Utopians. | |
| | Eccentrics. | |
| | Mixed conditions. | |
| Emotional states | Prostitutes. | |
| | Criminals. | |
| | Unstable. | |
| Neuralgia | External. | |
| | Internal. | |
| Psychoses | Acute insanity. | Symptomatical. |
| | | Idiopathic. |
| | | Alcoholic. |
| | Imbecility. | |
| | Idiocy. | |
| | Chronic insanity. | General insanity. |
| | | Dementia |
| | | Partial insanity. |
| Special and general conditions of the senses | | Congenital blindness. |
| | | Anæsthesiæ. |
| | | Deafness. |
| | | Hyperæsthesiæ. |
| Rachitis. | | |

For the purpose of comparison, I present from my own work[1] a table prepared from a dental stand-point independently thirty years after Moreau. There is a close similarity; the differences are only such as must occur in the course of three decades.

---

[1] Etiology of the Osseous Deformities of the Head, Face, Jaws, and Teeth.

TABLE B.

| | | |
|---|---|---|
| Cerebral . . . . | Ethical degeneracy. | Crime. |
| | | Prostitution and sexual degeneracy. |
| | | Moral insanity, pauperism, and inebriety. |
| | Intellectual degeneracy. | Intellectual degeneracy. |
| | | Paranoia. |
| | | Adolescent insanity. |
| | | Periodical insanity. |
| | | Hysteria. |
| | | Epilepsy. |
| | | Neurotics. |
| | | Genius. |
| | | Idiocy. |
| | Sensory degeneracy. | Deaf muteism. |
| | | Congenital blindness. |
| | Nutritive degeneracy. | Lymphoid degeneracy. |
| | | Tissue instability. |
| | | Plural births. |
| | | Bleeders. |
| | | Excessive fecundity. |
| | | Gout. |
| Spinal . . . . . . | Various congenital and hereditary disorders. | |
| Local reversional tendencies. | Jaws. | |
| | Cleft palate. | |
| | Teeth. | |
| | Primitive uteri | and allied male states. |
| | Cloacal conditions | and allied male states. |
| | Horseshoe kidney and allied states. | |
| | Cyclopean monstrosities. | |
| | Ameliacs and polymeliacs, club-feet, etc. | |
| | Plural mammæ. | |
| | Simian muscular and bony states. | |
| | Liver, and other organ reversions. | |

As the functions of the nervous system are not merely to govern motion and sensation, but to control nutrition, defects of the nervous system in this latter particular may produce acromegaly or other excessive and arrested developments of tissue. This may influence the body as a whole, producing a "Tom Thumb" or a Chestnut Street, Philadelphia, policeman, or may affect parts only, as the face from the lower border of the orbits down, including masticating surfaces of the upper molar teeth. The upper part of the body as far down as the pelvis may be normally developed, while the lower limbs and feet (frequently seen in club-feet) are undeveloped.

From this factor in nervous functions results the osseous expression of degeneracy in the defective classes, which expression, as will appear on analysis of the different classes, varies greatly. The prostitute, according to the researches of Pauline Tarnowsky, Solliër, Lombroso, and myself, present the most decided stigmata of all classes of degenerates. In them intrinsic rather than extrinsic causes play a large part. Even professional thieves are less decidedly a type of defectives. Their ancestry reveals a great proportion of syphilitics, alcoholics, sexual or other criminals, hysterics, and neuropaths.

Dr. Tarnowsky's researches show the professional prostitute to be a degenerate being, who is a subject of an arrest of development, tainted with a morbid heredity, and presenting stigmata of physical and mental degeneracy fully in consonance with her imperfect evolution. The degeneracy due to an imperfect organization in prostitutes principally appears in the frequency of skull deformities (forty-four and one-third per cent.) and deformities of the face (forty-two and two-thirds per cent.); by numerous anomalies of the ears (forty-two per cent.), and of the teeth (fifty-four per cent.).

The mental stigmata appear as a more or less marked intellectual feebleness, in a neuropathic condition, in a notable absence of moral sense.

Lombroso, on examination of fifty prostitutes, found exaggerated lower jaws twenty-six times, plagiocephalia twenty-three times, asymmetrical noses eight times, prominent zygomæ forty times.

In order to determine if such conditions existed in this country, I made, together with Dr. Harriet C. B. Alexander and Dr. J. G. Kiernan, some researches in the Chicago Bridewell. The inmates here are the least intelligent of any of the class. The researches are necessarily far from complete, owing to the difficulties under which such researches must be made in cases of persons with short-term sentences. They were, however, habitual cases, some being in the institution from time to time for twenty years, and most of them criminals. The number examined was thirty. All showed marked stigmata.

| | |
|---|---|
| V-shaped dental arches . . . . . . . . . . . . . . . . . . | 10 |
| Partially V-shaped dental arches . . . . . . . . . . . . . | 17 |
| Semi-V-shaped dental arches . . . . . . . . . . . . . . . | 7 |

| | |
|---|---|
| Saddle-shaped arches | 27 |
| Partially saddle-shaped arches | 10 |
| Semi-saddle-shaped arches | 10 |

The jaws in some cases were so badly deformed that frequently two deformities would be upon the same jaw.

| | |
|---|---|
| High and deformed zygomæ | 16 |
| Deformities of the face | 14 |
| Arrest of lower jaw | 1 |
| Arrest of bones of face | 4 |
| Abnormal noses | 6 |

The teeth were all irregular and badly decayed: sixteen showed discoloration of the enamel; nine, wearing away of enamel. Supernumerary tubercles were numerous, and eight were minus third molars in the upper jaw, and six in both jaws.

Hypertrophy of the jaw and alveolar process was common, and all had more or less tartar upon the teeth.

My object in dwelling upon this class of degenerates will be shown later on. Much more could be said on this subject, but enough stigmata has been demonstrated to show that this class is very low in the scale of human evolution.

For the purpose of comparison I present the findings of one particular variety in the other defective classes.

TOTAL DEFORMITIES IN THE JAWS OF IDIOTS.

| No. | . . | Normal. | Large jaw. | Protrusion lower jaw. | Protrusion upper jaw. | High arch. | V-shaped arch. | Partial V-shaped arch. | Thumb-sucking. | Saddle-shaped arch. | Small teeth. |
|---|---|---|---|---|---|---|---|---|---|---|---|
| 1977 | . . | 1095 | 152 | 92 | 159 | 318 | 129 | 236 | 31 | 207 | 71 |
| Per cent. . | | 55.3 | 7.6 | 4.6 | 7.9 | 16. | 6.5 | 11.9 | 1.5 | 10.4 | 3.5 |

TOTAL DEFORMITIES IN THE JAWS OF THE DEAF AND DUMB.

| No. | Sex. | Normal. | Large jaw. | Protrusion lower jaw. | Protrusion upper jaw. | High arch. | V-shaped arch. | Partial V-shaped arch. | Saddle-shaped arch. | Small teeth. |
|---|---|---|---|---|---|---|---|---|---|---|
| 1111 | Male. | 538 | 197 | 41 | 116 | 241 | 91 | 115 | 108 | 51 |
| 824 | Female. | 363 | 108 | 51 | 89 | 177 | 78 | 77 | 95 | 62 |
| 1935 | . . | 901 | 305 | 92 | 205 | 418 | 169 | 192 | 203 | 113 |
| Per cent. . | | 45.3 | 15.7 | 4.7 | 10.5 | 21.7 | 8.7 | 9.9 | 10.4 | 5.8 |

Two cases cleft palate.

TOTAL DEFORMITIES IN THE JAWS OF THE BLIND.

| No. | Sex. | Normal. | Large jaw. | Protrusion lower jaw. | Protrusion upper jaw. | High arch. | V-shaped arch. | Partial V-shaped arch. | Saddle-shaped arch. | Small teeth. |
|---|---|---|---|---|---|---|---|---|---|---|
| 107 | Male. | 53 | 8 | 9 | 10 | 20 | 4 | 3 | 6 | 7 |
| 100 | Female. | 52 | 8 | 7 | 5 | 18 | 3 | 6 | 5 | 3 |
| 207 | . . | 105 | 16 | 16 | 15 | 38 | 7 | 9 | 11 | 10 |
| Per cent. | . | 50.7 | 7.7 | 7.7 | 7.2 | 18.3 | 3.3 | 4.3 | 5.3 | 4.8 |

One case cleft palate.

INSANE TAKEN AS A WHOLE.

| No. | Sex. | Normal. | Large jaw. | Protrusion lower jaw. | Protrusion upper jaw. | High arch. | V-shaped arch. | Partial V-shaped arch. | Saddle-shaped arch. | Small teeth. |
|---|---|---|---|---|---|---|---|---|---|---|
| 430 | Male. | 394 | 10 | 4 | 2 | 18 | 12 | 29 | 3 | 5 |
| 270 | Female. | 226 | 8 | 6 | 4 | 26 | 14 | 18 | 9 | 2 |
| 700 | | 620 | 18 | 10 | 6 | 44 | 26 | 47 | 12 | 7 |

In one hundred professional men and expert mechanics, which come under the head of Geniuses, the following results were obtained:

DEFORMITIES OF THE JAW.

| | |
|---|---|
| V-shape | 12 |
| Partial V | 20 |
| Semi-V | 7 |
| Saddle | 21 |
| Partial saddle | 12 |
| Semi-saddle | 14 |
| Marked deformities of the face | 32 |

Other deformities of the head, ears, face, thyroid glands, etc., were very markedly developed among these degenerates.

From these tables it is observed that from sixty to ninety-five per cent. of at least one type of deformities occur among degenerates. As the ear varies enormously with the same class of persons, it is not surprising that, as Morel showed half a century ago, a demonstration which my own experience corroborates, the ear should be most frequently affected by degeneracy; next for the same reason come the jaws and teeth, and finally the head and face. Abnormal development of all is strong evidence of degeneracy. The data given are, with the exception of the geniuses, from inmates of public institutions. These considered as degenerates are the extreme wrecks,—wrecked by bad inheritance, but wrecked also by surroundings. Furthermore, degeneracy may be evident only in the return to bird and reptile states of the lever which produce hereditary gout, etc. Here the otherwise normal individual will present stigmata of degeneracy, while his less fortunate brother will be one of the defectives. These classes of apparently normal persons are numerous. One neurotic child receives good school training and lives a long life with only perchance a tendency to gloomy views of life. This was the case with Schopenhauer, the German philosopher. Several other children are wrecked by bad parental or school training. Some, however, survive even this, and their defect is shown only in an undue tendency to superstitious belief. This was the case with the novelist George Eliot, as Dr. Harriet C. B. Alexander has shown; another may inherit or acquire an inactive liver, like the author. As the extreme cases, therefore, only reach public institutions, the amount of degeneracy encountered by the dentist cannot well be over-estimated, since he sees from the nature of stigmata a larger number of relatively normal degenerates than the physician. Degenerate ears do not come to the otologist, since their function is apt to be normally performed. Degenerate teeth and jaws do not perform their functions normally, or, if they do, they interfere with the comeliness of the patient. It is not exceeding the mark to claim that from fifty to seventy-five per cent. of the dentists' patients are degenerates; hence it is well to examine the lesions of the tissues of the mouth, bearing in mind the fact that all such cases possess a defective nervous system.

Acromegaly and arrested development of the face, jaws, and teeth are almost invariably associated with degeneracy, although many cases are due to heredity or maternal worry and innutrition; yet cleft palate and harelip are the noticeable defects at birth. In all of these cases, with the exception noted, the extent of the deformity is not noticed until between the ages of twelve and eighteen years. These develop with the second set of teeth, and the position of the jaws to each other are thus expressed. Arrest of the upper jaw means a V or saddle arch; arrest of the lower, a saddle arch or an undeveloped chin. Acromegaly of the jaws and alveolar process are frequently noticed. Enlargement of the body of the jaws, of only one, or of the alveolar process, upper or lower, may occur. If the excessive development commences early, with the development of the second set of teeth (enlargement of the alveolar process is usually excited by the irritation of the erupting teeth), it will continue, and the teeth will be carried forward and laterally, causing space between. Frequently the enlarged alveolar process is only observed at the posterior part due to the eruption of third molars. In these conditions we frequently find small teeth when the germs and teeth are missing: they are invariably associated with a defective individual.

### ACROMEGALY.

Acromegaly may affect the body as a whole or parts. At the Dunning Asylum is an idiot whose body is nearly normal, but whose head, hands, and feet are twice the normal size. The jaws are large in proportion. A German leader in an orchestra has a head and face excessively developed, jaws and teeth very large. A Hebrew, who died of phthisis, had a lower lip so excessively developed that when drawn up it completely covered his nose. He possessed a very small upper jaw with V-shaped arch; the lower jaw was prognathous. Teeth were badly decayed, and there was an excessively large quantity of tartar. Such cases are frequently seen among our asylum patients, and are not uncommon in our daily practice.

The report of the work in the clinics of the Breslau Royal University includes a case of dental deformity in akromegalia in a mill-worker, always in good health, and from a healthy family. After serving his time in the army, in 1877, he noticed an enlargement of his hands and face, which grew for about three years, and then ceased. The patient is short, stout, rather heavily moving, though strong and strikingly pale, especially the lips and eyes, and the face puffed. The speech is slow, the thoughts inactive. The lower part of the face is strongly developed and the lower jaw and the upper lip protrude, allowing the forehead and skull to retreat. The lower jaw measures fourteen centimetres from chin to jaw-angle, and carries strongly developed soft and spongy lower lip. Its thickness is two and a half centimetres. The lower jaw projects at least one inch over the upper one. The distance between the lower wisdom-teeth is 6.8 centimetres, their connecting line with the lower central incisors measuring 4.9 centimetres. The distance between the wisdom-teeth in the upper jaw is four centimetres, and on their connecting line with the central incisors 3.2 centimetres. The teeth are quite decayed; those of the lower jaw do not strike their opposites. The upper jaw is normal. Especially curious appears the widening of the tongue; it is soft, its mucous membrane much wrinkled and folded; it is movable in all directions, though with some difficulty. The skin on the face is somewhat thick, faded, and weak. The facial bones show no softening. The neck is short and thick, but no swelling of the thyroid gland exists. The chest is well rounded, of normal volume.

### SUSCEPTIBILITY TO PAIN.

Pain susceptibility is, all things considered, less in the lower than in the higher types, but dread of pain has greater effect in the lower than in the higher. While degenerates suffer less from pain than the higher types, they dread it more, hence the frequent dangers in dentistry among this class. The dread acts like a shock.

Corpulent children, people suffering from disease, neurotic individuals, due to nervous strain, are the classes most susceptible to pain in dental operations.

### LIPOMATOSIS OR MORBID CORPULENCE IN CHILDREN.

Ebstein and other authorities have shown that obesity or excessive corpulence is an expression of the same trophic disorder as gout and rheumatism. Ere forty years, the period of involution especially, and ere puberty, such corpulence is attended by marked trophic change in the tissues of the body, whence the deficiencies already noted in fat children who readily fall victims to all sorts of microbic invasion, and whose cardiac and vasomotor state predisposes them to serious disorders.

### DEGENERACY OF THE NERVOUS SYSTEM AFTER FORTY YEARS OF AGE.

The period of life between forty and sixty is to a man a period of involution when certain functions are ceasing to be active factors in the life of the individual. The structures devoted to retrograde metamorphosis assume predominance, and the arterial system shows a tendency to fatty change. This most often occurs between forty-five and sixty, but cases at forty are far from infrequent. In both sexes at this period, as at puberty, there is marked tendency to nervous disease. The changes, of what may well be called the climacteric, are the results of deficient power of supplying nutrition rather than excess in utilization; hence the primary changes of the arteries and their secondary consequence in the bones and teeth. Sudden marked changes in the teeth are very likely to take place, such as abrasion, erosion, decay, and discoloration, which are noticed throughout this paper.

### DEGENERACY OF THE NASAL CAVITY.

Hypertrophy of the bones of the nose, mucous membrane, adenoid vegetation, and catarrh are associated with stenosis of the nasal cavity, deflection of the septum, arrested development of the superior maxilla, including mouth-breathing, V-shaped arch, and premature ossification of the bones of the base of the skull. One is not caused by the other, but all are due to the same cause,—degeneracy. Such conditions can be recognized at a glance, from the undeveloped appearance of the face.

### NYMPHOMANIA AND THE TEETH.

In every community exists a class of women, married and single, whose status in society would seem to indicate that their morals were of the best, but who in secret are demi-mondaines. Their characters are familiar among all classes. They present the same stigmata as those confined in public institutions. That prostitution is an inheritance is aptly illustrated in the history of Alphosine Plessis, idealized by Alexander Dumas in "Camille." Her paternal grandmother, who was a half prostitute, gave birth to a son by a country priest. This son was a peddler by trade. The maternal great-grandmother was a nymphomaniac, whose son married a woman of loose morals, by whom a daughter was born. This daughter married the peddler, and their child was Camille. She had undoubtedly the powerful perverted sexual instincts of her class. She died childless early in life from consumption. Children with this inherited taint, even if put into better surroundings, usually prefer debauchery when they grow up. Man is susceptible to many crimes, but woman diverges from the beaten path in the direction of the least resistance. These women, as a rule, possess fine features and are good looking. This is due, in many cases, to the fact that the bones of the face are arrested in development, the person retaining the childish features throughout life.

### TEETHING, GUM-LANCING, AND EXTRACTION.

Nothing satisfactory has been written upon the subject of teething explanatory of the causes of the systemic disturbances so immediately associated with physiological dental evolution.

Eruption of the teeth is a natural process, and, like child-bearing, should be accomplished with very little pain. The experience of physicians and dentists, however, demonstrates a different state of things. Not only do children often suffer intensely while the teeth are erupting, but convulsions and even death may ensue.

J. W. White[1] says, "It is rare for a child to pass through the period of dentition without more or less manifestations of suffering, and frequently there are serious and alarming disturbances of the health."

Close observation has shown that in a family of ten children only one passed through the evolutionary stage of teething without constitutional disturbance. A family of six children (five girls and one boy), had most alarming symptoms during the period.

One of the oldest theories as to the cause is that the tooth or teeth pressing against the gums produces the pain, another that the backward pressure of the teeth upon the superior or inferior nerve is the cause.

White further says, "In estimating, therefore, the mischief which may result because of a lack of accordance between the eruption of a tooth and the absorption of the tissues which impede it, we may imagine the sensitive nerve—which, when exposed by decay, is so intolerant of contact even with the atmospheric air—held between the bony socket and the sharp edge of the incomplete root by the backward pressure of the resisting gums, thus giving rise to a true toothache, comparable only to that exquisite torture which is experienced in after-life from an exposed and irritated pulp." These, together with the undeveloped nervous system, are supposed to be the cause of the systemic disturbances. It would seem, however, that since the nervous system is undeveloped, there is less sensitiveness to pain, and although the immediate cause is the erupting tooth, other and far greater predisposing causes than have as yet been advanced must be looked for.

Dr. Harriet C. B. Alexander,[2] in a very able series of papers upon the subject of "Acute Mental Symptoms in Children," has shown that the brain and nervous system of children are frequently unbalanced; a lack of equilibrium manifested in manners and actions.

From observation, I am convinced that those children who suffer with complications in teething are the victims of an inherited or acquired neurotic constitution; while the advancing tooth or

---

[1] The Mouth and Teeth.

[2] *Medical Standard*, April, May, June, 1893.

teeth is the exciting cause, the systemic complications are due to an unbalanced nervous system. On the other hand, a strong, healthy child, whose nervous system develops in harmony with the other tissues of the body, rarely suffers during teething. The children of the two families just mentioned were victims of inherited degeneracy. The proper thing to do in such cases is to assist the eruption immediately,—namely, lance the gum. This, however, is not infrequently followed by disastrous results,—excessive hemorrhage, and occasionally death, evidence of another type of degeneracy, the bleeder. Great care, therefore, should be exercised in ascertaining the general conditions of the child ere grave results appear.

In 1872[1] I was called to lance the gums of a puny, sickly, anæmic child. Although there was plenty of nourishment from the mother, the child did not flourish. I lanced the gums, but she died, despite all the family physician and myself could do to arrest hemorrhage.

The dentist not rarely comes in contact with bleeders in extracting teeth and removing tartar, and not infrequently death results. I have never had a death from extracting, but I have had very serious hemorrhages from cleaning and extraction; one case of which is here of interest. In 1870 a patient came to me to have his teeth put in order. As is customary, I first removed the tartar and cleaned his teeth, which I had some difficulty in doing because of hemorrhage of the gums. I was unable to do more at that sitting, owing to the excessive bleeding, so I dismissed him. At three o'clock the next morning I was sent for, and found the family physician had been all night trying to stop the hemorrhage. After five days and nights I finally controlled it. It probably stopped on account of the weak condition of the system. The patient, however, remained in bed two months in a very low state. I learned from his physician that he had had two hemorrhages of the urinary tract, presumably of the bladder, and that he was consumptive. When he recovered, his physician sent him to California, where I lost trace of him. It would seem to the average dentist, reading an account of a death from hemorrhage from extraction, that there must be carelessness or neglect on the part of

---

[1] While each point herein made is illustrated with but from one to three cases in practice only, it could easily be supported by numerous others from the author's note-book.

the operator. I can assure you that the dentist who has a bleeder under treatment deserves the sympathy of his fellow-practitioners.

### ABRASION, EROSION, AND DISCOLORATION OF THE TEETH.

In the "American System of Dentistry," Dr. Black, after discussing this subject in thirteen and a half pages, frankly admits that we know very little about it. The literature of this subject proves that he is correct. Much has been written, but nothing developed. By close observation, however, a little light may be thrown upon the subject. Abrasion of the teeth of the ancient Egyptians, Peruvians, Mound Builders, Cliff Dwellers, Indians, and all those people who live upon coarse food is one thing, and that observed in civilized races to-day is another. Both are the result of friction, but one is a physiological process, the other pathological. One is a gradual wearing away of the teeth of strong, healthy, robust persons in their struggle for existence, the other is the result of tooth-softening due to a degenerate nervous system. People exist to-day, however, whose teeth wear away like the ancients.

Certain able dentists claim that abraded teeth are harder and more dense than ordinary teeth; my observations show that they are much softer, and that they cut easier than normal teeth.

Abrasion and erosion of the teeth commence more frequently after the fortieth year than at any other time. Occasionally, however, they are connected with the first set of teeth and at different periods through life. Sometimes they are spontaneous and make their appearance almost immediately after illness, after severe mental strain, grief, or at any period in neurotic children. In connection with abrasion and erosion due to pathological conditions there is almost invariably present discoloration of the enamel and dentine. Occasionally erosion and abrasion appear without discoloration and *vice versa.*

Discolorations appear frequently early in life upon the first and second teeth after severe illness, and frequently appear in life after the fortieth year. The discolorations are often seen in small spots upon one tooth, confined entirely to one tooth, or all the teeth on both jaws will become involved. Erosion will affect the whole surface of one tooth, or only in spots, in one or in all the teeth. The surface is usually polished and apparently hard, while underneath, the enamel and dentine are soft. The tooth is less sensitive and the pulps have receded. Whenever discoloration is observed, the tooth is always softened; the discolored part is unusually so when all or nearly all the teeth are involved. The enamel can be removed from the dentine with ease, and the dentine can be cut like horn without pain. Oftentimes the crowns will be partially or entirely worn away. There is usually no sensitiveness in the dentine, the pulps have receded, and the pulp-chamber is filled with uncalcified dentine. Cases of this nature illustrated in the "American System of Dentistry" (pages 415 and 416, figs. 106 and 107) by Dr. Barrett, of Buffalo, N. Y.

Dr. D. B. Freeman, of Chicago, has remarks of a similar case cited in the "American System of Dentistry:"

"The patient was a medical student, twenty-six years of age. The enamel was totally lacking on all of his teeth except the second and third molars, and even there it was deficient and imperfect in places. All the crowns were very much worn, those anterior to the molars being level with the gum line. They were not then, nor had they been, sensitive. He could not remember when they were in any other condition or in appearance different from that when they were presented. He said his ancestors for three generations, on his father's side, had this peculiar deficiency of crown structure, and his three brothers and two sisters presented this same condition."

I have had seven similar cases. The abrasion, however, was not quite so extensive in any of them. One case deserves mention: Mrs. A., an American, is a born degenerate, small in stature, small long bones, small head, face, and jaws. Her first teeth were soft, decayed early, and were lost before the time for the eruption of permanent teeth, which teeth came in in fairly good shape, although a few small fillings had to be inserted. She married at twenty. A still-born child was the result of a two years' marriage. At this time her teeth began to soften; although her appetite is good, she is tired all the time, and her nervous system has become depressed. Her teeth were all filled in good shape at the time of her marriage. The enamel softened and disintegrated, leaving the fillings standing attached to the dentine. The enamel can be removed in large pieces from the dentine, showing that there is but little attachment. The enamel is like chalk and can be removed readily. The dentine in places where exposed is but slightly sensitive. It is discolored and can be ground down like horn. The bicuspids and molars, upper and lower, are affected. The incisors and cuspids, while they have not disintegrated, have grown quite dark and are soft like the other teeth. There is no decay, though the teeth have soft spots in places. This wearing away is upon the grinding surfaces, although in most places friction does not come in contact with them. A similar case is cited in my work,[1]—Case VII., Fig. 60, p. 240.

In my asylum investigations for the past twenty years, I have not failed to note the peculiar condition of the teeth in certain neuroses, particularly paretic dements and spinal lesions such as cord injuries and locomotor ataxia. I have had eighteen ataxic patients in my private dental practice. Three hundred and thirty-two dements all showed more or less abrasion. Forty-seven ataxic patients all showed abrasion. Post-mortem examination reveals tissue-change of the nerve-structure and also of the arteries. The trophic centres become disordered and body nutrition is impaired. Not only are the nails, hair, and skin affected, but the teeth soften and are worn away.

In most ataxic patients and many dements, marked cupping out of the crowns is observed. In other forms of insanity we find a large percentage of abrasion as in cases due to syphilis and other diathetic states.

In Egyptians, Peruvians, Mound Builders, Cliff Dwellers, and a few of our people whose central incisors do not lap but meet squarely, the motion of the jaws in mastication is quite different from those whose incisors lap. Abrasion takes place from the rotary motion of the jaws, "mill-like"; not only are the cusps ground off smooth, but the teeth are gradually worn away until the gums are reached. I have observed this condition in the first set of teeth. When this condition takes place, the dentine about the pulp-chamber and the secondary dentine is invariably discolored; we do not find erosion in this class of patients, nor do we find it to any extent in asylum cases.

In the pathological cases, however, they are quite marked. It seems to be associated with our modern style of living and habits,—that is, first softening, and then friction. Mental strain, disorder of the arterial and nervous systems from diseases, and a general change in the system are the most frequent causes of abrasion, erosion, and discoloration of the teeth after forty years of age.

Two cases are of interest and identical: Mr. D., sixty-four years of age, Mr. M., sixty years, both lawyers of good standing, both patients for twenty-one years. The teeth of both gentlemen were strong and well developed, and required very little attention, except the removal of tartar, until about ten years ago, when they began to soften and decay; within the last three years it was with

---

[1] Etiology of Osseous Deformities of the Head, Face, Jaws, and Teeth.

difficulty that I was able to preserve them. Mr. D. died suddenly, September, 1892, from aneurism of the aorta. Mr. M. died September, 1895, of the same lesions. Post-mortem revealed atheroma of the arteries, which had been going on for some time. The year before Mr. M. died the teeth cut like soft cheese and decay went on continually. Although I had taken excellent care of his teeth, I filled twenty-three cavities two months before death. Abrasion of the teeth commenced when the gentlemen were about fifty years of age, and had gone on to a great extent at the time of death. It will be observed that I make no distinction except locality and color in the three conditions, abrasion, erosion, and discoloration of the teeth, all being the result of disorder of the nervous system.

We do not have to go far to corroborate the views herein expressed, that these lesions are due to trophic changes.

### ABRASION, EROSION, AND DISCOLORATION.

The bone changes so prominent a trophic feature in locomotor ataxia were necessarily, as Duchenne de Boulogne pointed out two decades ago, accompanied by changes in the teeth. At the meeting of the French Association for the Advancement of Science, August 30, M. T. David read a paper upon lesions of the teeth in locomotor ataxia, based upon the analysis of a single case. The most important of the conclusions arrived at were: The alterations consisted of a rapid decay of the anterior part of the crown of almost all of the teeth. The altered substances assumed the consistence of touch-wood of a reddish color. The enamel still retained its polish but not its hardness. Beneath those parts of the pulp had been produced a new layer of secondary dentine, and in most of the front teeth the pulp-cavity was filled up. These alterations had nothing in common with caries, and must be referred to nutritive disturbance resulting from the lesion of the central nervous system. The changes are analogous to those which have already been observed in the nails in locomotor ataxia. They then establish a pathological relationship between organs already connected by a common epithelial origin. Locally these alterations recognize for their immediate cause a functional disturbance or a lesion of the dental pulp. The atrophy shown to exist would be quite comparable to that observed in the eye under similar circumstances. What is true of locomotor ataxia is true of its closely allied nervous disease, paretic dementia, or paresis.

In sixteen ataxic patients under my care for dental services, all possessed marked abrasion with considerable cupping. Out of twenty-nine cases of locomotor ataxia in our public institutions, twenty-eight had marked abrasion, and one was not sufficiently advanced to call it a decided case. The physician in charge was not quite certain of his diagnosis.

I have observed three cases: One, a Pole twenty-six years of age, had good sound teeth until his back was broken. When I saw him, eighteen months after, all of his teeth showed marked abrasion and cupping. Another, an Austrian forty-five years of age, had his back also broken. Both men were paralyzed in the lower limbs. The latter also showed marked abrasion. A very interesting case is that of Lee Soy, a Chinaman. He has lived in America twenty-seven years, and has had locomotor ataxia eight years, the result of syphilis. Abrasion presents itself on all the teeth. The molars and bicuspids are cupped out.

Dr. Edward F. Keefe, of Chicago, who has quite a large practice among Hebrews, has a large number of patients suffering from erosion. Although he is preparing a paper upon this subject, he has kindly allowed me the use of some of his cases for this paper.

Mr. E., forty-six years of age, of nervous temperament, suffered with indigestion. All the anterior teeth, upper and lower, badly affected; had had this condition seven years and seven months.

Mr. S., forty-five years of age, health good; six years ago had nervous prostration. At this time the erosion took place. Is now in good condition. The erosion has stopped.

Mr. H., forty years of age; nervous temperament. Can't remember when erosion began; has just noticed it.

Mr. S., forty-six years of age. Duration, five years. No history. Upper left incisor and cuspid.

Mr. M., thirty-three years of age. Duration, one year. Nervous temperament. Upper right and left bicuspids and one molar.

Mr. B., forty-one years of age. Duration, two years. Nervous temperament. Upper and lower incisors and bicuspids.

Mr. M. No history; sixty-five years of age. Upper incisors and right cuspid.

Mr. K., sixty years of age. No history. Lower left incisor and cuspid. Disease ceased.

Mr. G., thirty-nine years of age. Duration, four years. Upper centrals, left lateral, left lower second cuspid.

Mr. D., forty-five years of age. Nervous temperament. Duration, five years. Upper and lower incisors. No history.

Mr. M., sixty-five years; sick twenty years.

Mr. P., aged forty-five years. Nervous temperament. Duration, five years. Upper right bicuspid, left upper bicuspid, lower left cuspid and first and second bicuspid.

I record these cases just as they were given to me on examination tablets, with the teeth marked showing erosion. Since this class of individuals are rich in the inheritance of all the ills that flesh is heir to, and since nervous disorders are very common among these people, they furnish a rich field for the study of erosion, abrasion, and discoloration. If it were possible to study each case, we should, no doubt, find that all had some trophic change at the commencement of this lesion. In three hundred and forty-three paretic dements and general paralytics, every patient showed abrasion to a more or less marked degree. This is to be expected, since there is a similarity in the nature of the disease.

Abrasion or erosion in a number of cases has been shown to progress rapidly and then cease altogether, or it will progress slowly and continue through life. Abrasion and erosion are frequently observed in the same mouth and on the same teeth.

### DECAY OF THE TEETH.

Admitting that the immediate cause of decay of the teeth is micro-organisms which must always have the same food to thrive upon, regardless of surroundings (since dentists are unable to effect a permanent cure by simply filling the cavity), other causes far more reaching in these destructive elements than those already laid down in text-books must be looked for. Clinical history and observation can accomplish a great deal in this direction. First, in regard to the general formation of enamel and dentine.

Text-books, in a gingerly fashion, allude to a predisposing cause, that of heredity in producing faulty formation of the teeth. In my opinion, acquired causes like worry, starvation, and fright of the mother, starvation, and improper assimilation of food after birth produce more stigmata than any one cause. Two factors enter into the cause of degeneracy: first, heredity; secondly, disorder of the trophic centres, due to local causes. These are the predisposing causes of faulty tooth-development, producing pits, furrows, and interglobular spaces.

Patients, no matter at what age, when attacked by a constitutional disease, suffer from decay to a greater extent than at any other time. The teeth of pregnant women decay more at that period than at any other time, and not infrequently a tooth, or even all the teeth, will be lost in six months to one or two years. This happens when complications arise. Fill the teeth of a young man or woman, and after a year at college or boarding-school, in many cases they return with cavities in nearly every tooth. These conditions are familiar to every practitioner, and illustrations are not necessary. Foreigners, such as Swedes, Norwegians, etc., coming to this country, experience such a change in climate and food that they take on the constitutional diseases and various stages of insanity much more readily than in their native land. The teeth decay much more rapidly than in any other class of individuals. These changes can be accounted for only by change that takes place in the trophic centres.

People over forty years of age not infrequently have decay attack their teeth, and it will be as impossible to protect them from decay as it is to protect the first teeth of a neurotic child. The two cases, those of Mr. D. and Mr. M., already alluded to are good examples of decay of the teeth from physical degeneracy. One case, due to overwork, will not be out of place here: Mrs. J., married, forty-eight years of age, a professor in Women's Medical College, Chicago, had, until three years ago, a very fine set of teeth. She is a large, healthy, well-developed woman. Overwork at the World's Fair and her college duties caused rapid decay of the teeth to the extent that she nearly lost the left lateral incisor. It was a question whether to build it out with cement or adapt an artificial crown. By taking a trip abroad and treatment, she has recovered her strength, and the mouth and teeth are in a much healthier condition.

The effect is, that the assimilation powers are lost, and as a result, the developing tissues are improperly formed, and if fully developed they degenerate. It can, therefore, be readily understood how, at any time in life, whether from inherited or acquired taint, constitutional disease, or overstrain from mental work, trophic changes in the nervous system will produce changes in tooth-structure. If the food products which sustain the micro-organisms be present, decay will take place in proportion to the power of resistance of the tooth on the one hand and the quality and quantity of the germs on the other. Decay of the teeth takes place in degenerates more readily and rapidly than in healthy individuals. This fact is easily demonstrated by examining the mouth of those confined in asylums. This is especially true of professional prostitutes. Dentists need not go out of their offices to demonstrate the fact. In classifying patients, the difference can be readily seen.

Trophic changes due to constitutional disease as illustrated by so-called Hutchinson teeth, pits and furrows upon the enamel, and malformation of teeth, are familiar to every dentist. Spontaneous death of the pulp, from fillings and other causes, which so frequently occurs in some mouths, is always observed in the mouths of degenerates.

Teeth decay more readily in the mouths of those whose jaws are arrested in their development than in others, a marked illustration of the power of degeneracy.

Softening of the teeth, abrasion, erosion, and discoloration, even to the extent of the crowns being entirely worn down without decay, may occur, the culture medium for micro-organisms not being present. Trophic disorders themselves furnish pabulum for micro-organisms.

In order to show how degeneracy will occur in families, I herewith present the histories of three families, citing such stigmata of degeneracy only as are of interest to us practitioners of dentistry.

Case I.—An American professional man married a Welsh woman. Both have marked facial stigmata. The man's jaws are arrested in development. The alveolar processes are undeveloped, bringing the jaws close together. The teeth are irregular, and (at the age of fifty-three) are loose on account of deposit of calcic salts. The upper jaws of the wife are small. The teeth are irregular. There is partial saddle-arch. At the age of forty-eight she had lost several teeth from decay and calcic deposits. Her ancestral history had a strong degenerate (insane) taint.

Six children were the offspring of the marriage. The oldest (a twenty-year-old girl) is of a nervous temperament, and short in stature. She has arrest of the facial bones and decided prognathism of the upper and lower jaw. The teeth are small. There are large spaces between the incisors, bicuspids, and molars. The upper and lower cuspids are undeveloped,—evidently from the density of the alveolar process,—although they are in position in the jaws, with plenty of room.

An eighteen-year-old girl, the second child, has still more decided stigmata. There is a full forehead, long slender nose, arrest of upper and lower jaw, partial V-shaped arch. The teeth are regular, but badly decayed. There is a large thyroid gland. The patient is a tall, slim girl, with long, slender small bones, dry skin and is poorly nourished. She is without principle or stability, and easily led. She became pregnant through (it was ascertained by her parents) some one of six boys who had been intimate with her. A young man married her on payment of a large sum, assumed the paternity of the child, and moved to a distant part of the country.

The third child is a sixteen-year-old boy.

Arrest of development of the entire body, rachitic, arrest of the bones of the face, jaws small, teeth soft, chalky, and badly decayed.

The fourth child (a fifteen-year-old girl) is phthisical, and has a spinal lesion. She is slender; she is unable to go to school on account of her irritability. She cries on very slight provocation and sleeps poorly. The jaws are small. There is arrest of the anterior upper and lower jaw. The left inferior second biscupid is undeveloped. There is a saddle-shaped arch. The temporary superior cuspids are in place. There are no signs of the permanent teeth. There is much tartar on upper and lower jaws. The gums are puffy and hemorrhagic. There is seeming sensibility to sudden unexpected pain.

The fifth child (a ten-year-old girl) is of nervous temperament and somewhat precocious. The forehead is very prominent. The vault is high, with long development of the median suture. Most temporary teeth are in place, but badly decayed. The first permanent molars have large crown fillers, and there are yellow-gray patches on these teeth.

The sixth child (seven years old) does not yet display any stigmata. The jaws are so developed that the incisors strike square, and abrasion is quite marked upon the first teeth. The cuspids and temporary first molars are also worn away perceptibly. The lower permanent first molars are in place. Their enamel is discolored. There is no appearance of upper first molars. The child (a boy) has an unusually large head and jaws for his age, evincing acromegalic indications. The probabilities are that he will be marked by prognathism. There is a marked ridge of the median line of the roof of the mouth.

Case II.—Three years ago a sixty-four-year-old American died. After his death he proved poor, in lieu of being wealthy, and was buried by his friends. He had indulged his family in expensive tastes. He had a rather high, dolicephalic head. Eyes small and set close together; long aquiline nose, long Morel ears, with Darwin tubercles; high cheek-bones; small upper jaw, V-shaped arch; long slim lower jaw; arrest of the alveolar process; teeth close together, the upper teeth badly decayed, with large collection of tartar. Three molars and one bicuspid on the lower jaw, one molar and two bicuspids on the upper, are lost from pyorrhœa. The remainder of the teeth were getting quite loose. The thyroid gland was arrested in its development. The wife, now living, aged fifty-nine, is rather a fine-looking American woman. The stigmata are not noticeable. The jaws are large and well-developed. Teeth were large and sound until fifteen years ago, when they began to decay. Tartar began to deposit at the time. About four years ago I extracted the lower incisor with my fingers. The teeth are decaying rapidly from age, grief, and worry for the conduct of her children. She had had four, of whom three are living. The oldest (a daughter, married sixteen years ago) has had nervous sick-headache all her life, with occasional epileptic attacks. She has a violent temper at times. She has no idea of the value of money, and resembles her father as far as stigmata are concerned. There is a V-shaped arch, and hypertrophy of the alveolar process. The pulps of her teeth die under gold fillings. All those I have filled I have had to extract on account of the persisting destruction of pulp and the possible formation of abscesses, which cause much pain. She has had no children because of an undeveloped uterus. She has led a questionable life, for which reason her husband committed suicide only a year ago.

The second oldest (a daughter) married twenty years ago, as a result of which two daughters were born. Before the birth of the second daughter her husband ran away because of her immoral conduct. Her features are regular, except that the eyes are small and set close together, like her father's. The jaws and teeth are well developed. The teeth are soft, and decay rapidly. Discoloration of the molars and bicuspids, with abrasion and erosion (only slightly noticeable at present).

The third (a boy, now twenty-nine years of age) went through the public school, and is a fair scholar. He has never done a day's work, and leads a debauched life, and formed the opium-cocaine habit. He has been in the State Insane Hospital and a number of private institutions. He is a hopeless wreck physically. Although his intentions are good, he has no will power. He has a small head, receding forehead; small eyes, closely set; high cheek-bones; prognathous jaws; excessively large teeth, with very prominent cuspids, upper and lower. His head resembles very much that of the anthropoid ape. The teeth are very irregular and badly decayed. Supernumerary cusps upon molars; there are deposits of tartar.

The oldest grand-daughter (eighteen years old) is a tall, slender neurotic; full forehead, small nose, large eyes, cheek-bones prominent, upper jaw well developed, lower arrested; dental arches are normal. Pulps die under fillings. I have difficulty in preventing abscesses forming.

The youngest grand-daughter (fifteen years old) is also a neurotic, and resembles her mother. Her teeth are badly decayed, although they are regular. Pulps die readily. She has large thyroid gland. This girl has inherited the habit of biting her finger-nails (which are badly deformed) from her mother and grandfather.

This is a degenerate symptom, according to Bertillon and others.

Case III.—A girl, ten years ago, left her home one morning to attend school. She met a man with whom she was very well acquainted. They were married in the afternoon. Girl twins were the result after two years of married life. Both husband and wife were degenerates mentally and physically.

The conduct of the wife was such that a year ago the husband procured a divorce, he retaining the children. Her face is small, eyes small, set close, prominent cheek-bones, partially V-shaped arch. Teeth are irregular and badly decayed. She has large thyroid gland. Both are from old degenerate families.

In conclusion I wish to say that, in order to better understand the lesions of the oral cavity, we must obtain a better knowledge of the physiology and pathology of the body. I will reiterate what I have said many times,—that any advancement made in our specialty must be accomplished through a broad, liberal medical education.

